# Secreted frizzled-related protein 5 protects against renal fibrosis by inhibiting Wnt/β-catenin pathway

**DOI:** 10.1515/med-2024-0934

**Published:** 2024-03-28

**Authors:** Dai Deng, Dongli Tian, Yahui Wang, Yu Bai, Zongli Diao, Wenhu Liu

**Affiliations:** Department of Nephrology, Beijing Friendship Hospital, Capital Medical University, 100050, Beijing, China; Department of Emergency, China Rehabilitation Research Center, Beijing, China; Department of Nephrology, Beijing Friendship Hospital, Capital Medical University, 95 Yong’an Road, Xicheng District, 100050, Beijing, China

**Keywords:** secreted frizzled-related protein 5, renal fibrosis, epithelial–mesenchymal transition, Wnt/β-catenin pathway, chronic kidney disease

## Abstract

Renal fibrosis (RF) is an important pathogenesis for renal function deterioration in chronic kidney disease. Secreted frizzled-related protein 5 (SFRP5) is an anti-fibrotic adipokine but its direct role on RF remains unknown. It was aimed to study the protective effect of SFRP5 against RF and interference with Wnt/β‐catenin signaling pathway for the first time. First, the therapeutic efficacy of SFRP5 was evaluated by adenovirus overexpression in rats with unilateral ureteral obstruction (UUO) *in vivo*. Thirty-six rats were randomly divided into the sham, UUO, and SFRP5 (UUO + Ad-SFRP5) groups. Half rats in each group were selected at random for euthanasia at 7 days and the others until 14 days. Then, the transforming growth factor (TGF)-β1-induced epithelial–mesenchymal transition (EMT) was established in HK-2 cells *in vitro*. The cells were divided into four groups: the control group, the TGF-β1 group, the TGF-β1 + SFRP5 group, and the TGF-β1 + SFRP5 + anti-SFRP5 group. The makers of EMT and Wnt/β‐catenin pathway proteins were investigated. In the UUO model, expression of SFRP5 showed compensatory upregulation, and adenoviral-mediated SFRP5 over-expression remarkably attenuated RF, as demonstrated by maintenance of E-cadherin and suppression of α-smooth muscle actin (SMA). *In vitro*, SFRP5 was shown to inhibit TGF-β1-mediated positive regulation of α-SMA, fibronectin, collagen I but negative regulation of E-cadherin. Furthermore, SFRP5 abrogated activation of Wnt/β-catenin, which was the essential pathway in EMT and RF pathogenesis. The changes after a neutralizing antibody to SFRP5 confirmed the specificity of SFRP5 for inhibition. These findings suggest that SFRP5 can directly ameliorate EMT and protect against RF by inhibiting Wnt/β-catenin pathway.

## 1 Introduction

It is widely recognized that all the main causes of chronic kidney disease (CKD) share the common pathogenesis of progressive injury as a fibrosis reaction [1]. Renal fibrosis (RF) is one of the most important pathological processes from CKD to end-stage renal disease (ESRD) caused by various reasons, in which the most obvious pathological feature of kidney is the deposition of collagen and related matrix in renal interstitial [2]. The epithelial–mesenchymal transition (EMT) in tubular epithelial cells is known to contribute to the pathogenesis of RF, which progresses ultimately to ESRD [3]. Thus, EMT inhibition may improve RF and delay the gradual loss of renal function.

The studies on pathogenesis indicate that Wnt/β-catenin plays a pathogenic role in RF which can be ameliorated by a Wnt antagonist [4,5]. Wnt protein promotes β-catenin-mediated signal after binding through complex receptors (coiled Frizzled and low-density lipoprotein receptor related protein-5/protein-6) [6]. The continuous activation of intracellular β-catenin signal has become an important pathogenesis of RF, while blocking the signal may be beneficial to alleviate fibrogenesis [7]. Secreted frizzled-related proteins (SFRPs) inhibit Wnt/β-catenin through binding and inactivating Wnt ligands, thus acting as Wnt antagonists [8]. SFRP5 is a secreted adipokine in SFRPs family, which may inhibit canonical or non-canonical Wnt signaling [9]. SFRP5 has a protective effect on liver fibrosis and oral submucous fibrosis against fibrotic disease [10,11]. Recent research suggests that indoxyl sulfate promotes RF by inducing SFRP5 DNA hypermethylation followed by activation of Wnt/β-catenin [12]. This finding has provided a novel insight into RF pathogenesis in CKD. However, whether SFRP5 can directly ameliorate EMT and protect against RF by inhibiting the Wnt/β-catenin axis is unknown.

The aim of this study was to determine the antifibrotic effects of SFRP5 on the unilateral ureteral obstruction (UUO)-induced tubulointerstitial fibrosis in a rodent model and EMT induced by transforming growth factor (TGF)-β1 in human proximal tubular epithelial cells. We also explored whether the direct effect of SFRP5 is mediated by interference with the canonical Wnt/β‐catenin by investigation of signaling molecules *in vitro*.

## 2 Materials and methods

### 2.1 Animals and experimental design

Eight-week-old male Sprague-Dawley rats (weighing approximately 170–190 g) were obtained from the Institute of Laboratory Animal Science (Chinese Academy of Medical Sciences, Beijing, China). As in a previous study [13], 36 rats were randomly divided in equal numbers into the sham group (sham + control adenovirus [Ad-GFP]), the UUO group (UUO + Ad-GFP), and the SFRP5 group (UUO + Ad-SFRP5). UUO was created surgically via laparotomy, after which rats in the sham group and UUO group received an intravenous renal injection of 1.0 × 10^10^ plaque-forming unit (PFU) control adenovirus, and those in the SFRP5 group received an intravenous renal injection of 1.0 × 10^10^ PFU adenovirus-SFRP5 [14,15]. Using a previously described method for creation of UUO [13], the same amount of adenovirus was re-administered 3 days later by tail vein injection. Half of the rats in each of the three study groups were selected at random for euthanasia at 7 days and the other half was euthanized at 14 days. Blood samples and kidney tissues were then collected from rats for a variety of analyses. All animal procedures were performed in compliance with local institutional and governmental regulations and approved by the Animal Experimentation Ethics Committee of Beijing Friendship Hospital.

### 2.2 Cell models and treatments

Human proximal renal tubular epithelial (HK-2) cells were obtained from the American Type Culture Collection (Manassas, VA, USA) and cultured in matching medium as before [13]. After starvation of serum for 12 h, the cells were incubated with SFRP5 (100 ng/ml; R&D, Minneapolis, MN, USA) or SFRP5 plus neutralizing anti-SFRP5 (1 μg/ml, R&D) in the absence or presence of TGF-β1 (2 ng/ml; R&D). Immunoglobulin G (1 μg/ml, R&D) was added for anti-SFRP5 as an additional control. Fresh medium was replaced every 48 h. Cells were harvested for a variety of analyses.

### 2.3 Detection of RF and EMT

The major pathological changes in RF are replacement of normal nephrons by numerous fibroblasts and accumulation of extracellular matrix (ECM), including fibronectin and collagen, resulting in glomerular sclerosis, tubulointerstitial fibrosis, and ultimately loss of renal function. Increased ECM promises that fibrosis is inevitable following excessive collagen deposition. Collagen I and fibronectin were detected by Western blot (WB) to evaluate deposition of ECM and reflect fibrosis. Fibrosis was also detected directly *in vivo* using Masson’s trichrome staining (MTS).

### 2.4 Semi-quantitative analysis of RF

To assess the RF extent, kidney sections embedded by paraffin were subjected to MTS [16]. The MTS sections were viewed under an Eclipse E600 epifluorescence microscope fitted with a digital camera (Nikon, Melville, NY, USA). The fibrosis severity was defined as the percentage of areas positive for MTS and analyzed by LabWorks image acquisition and analysis software (Ultra-Violet Products, Cambridge, UK). Five randomly chosen non-overlapping fields from each sample were analyzed.

### 2.5 WB analysis

Kidney tissue and cell samples for WB detection were prepared according to the aforementioned method [13]. Primary antibodies (all sourced from Abcam, Cambridge, MA, US) and dilutions were as follows: anti-glyceraldehyde-3-phosphate dehydrogenase (GAPDH; 1:5,000, ab181602), anti-SFRP5 (1:100, ab230425), anti-α-SMA (1:1,000, ab5694), anti-E-cadherin (1:500, ab40772), anti-fibronectin (1:1,000, ab2413), anti-collagen I (1:500, ab138492), anti-β-catenin (1:1,000, ab6301), anti-phospho-β-catenin (S37) (1:1,000, ab75777), anti-histone H3 (1:1,000, ab1791), and anti-Snail (1:500, ab216437).

### 2.6 RT-qPCR

RNA isolation and RT-qPCR steps were performed by standard procedures, using Power SYBR Green PCR Master Mix (Applied Biosystems, Waltham, MA, USA) on an ABI 7500 sequence detection system. Table 1 lists the primers and then target genes’ mRNA levels were calculated by taking GAPDH mRNA for normalization.

**Table 1:** Primers used for RT-qPCR

Primer	Sequence
SFRP5	F 5′-ACTTTGAATGCCGTGAA-3′
	R 5′-AATCCAGTTGGTGAGCC-3′
α-SMA	F 5′-ACTGCCTTGGTGTGTGACAA-3′
	R 5′-TCCCAGTTGGTGATGATGCC-3′
E-cadherin	F 5′-TCATGAGTGTCCCCCGGTAT-3′
	R 5′-TCTTGAAGCGATTGCCCCAT-3′
c-Myc	F 5′-CTACTTGGAGGAGACATGGTG-3′
	R 5′-TGGAGGTGGAGCAGACG-3′
Cyclin D1	F 5′-AGTAGCAGCGAGCAGCAGAGT-3′
	R 5′-TTCATCTTAGAGGCCACGAA-3′
GAPDH	R 5′-TGGAGGTGGAGCAGACG-3′
	F 5′-GAGGCTCTCTTCCAGCCTTC-3′

### 2.7 Immunohistochemistry

Paraffin-embedded sections of renal tissues and immunohistochemical staining were carried out according to standard procedures. The primary antibody for immunohistochemistry was rabbit polyclonal to anti-SFRP5 (5 µg/ml, ab230425, Abcam) and using PBS to replace as negative control. The slides were incubated with primary antibody at 4°C overnight, followed by incubation with enzyme-conjugated secondary antibodies and development with diaminobenzidine (DAB). The above-mentioned inverted microscope was used to observe the slides and images were collected from randomly selected fields.

### 2.8 Immunofluorescence

Briefly, cells grown on cover slips were fixed with 4% paraformaldehyde followed by incubation with primary antibodies against α-SMA (1:100, ab5694, Abcam) or E-cadherin (1:100, ab40772, Abcam). The paraffin sections of kidney tissues were performed by multiplex staining and multispectral imaging to detect the EMT markers. The slides were incubated with antibodies of α-SMA and E-cadherin sequentially and then incubated with the horseradish peroxidase-conjugated secondary antibody using tyramide signal amplification. Nuclei were stained with DAPI (Sigma-Aldrich, St. Louis, MO, USA) after antigen labeling. The scans of the slides were obtained by the Mantra System (PerkinElmer, Waltham, MA, USA), and combined as a single stack image. The spectral library was established using inForm image analysis software (PerkinElmer). The final images were obtained after removing the autofluorescence.

### 2.9 Immunoprecipitation

Cells were incubated with 2 ng/ml TGF-β1 in the presence or absence of 100 ng/ml SFRP5 for 72 h at 37°C. Immunoprecipitation was carried out with a Pierce Classic IP kit (Thermo Scientific, Rockford, USA). In short, cell lysates were incubated with 4 µl antibody for Wnt5a (Millipore, Merck, Germany) or IgG (Santa Cruz Biotechnology, Inc., Oregon, USA) at 4°C overnight. After that, the precipitated complexes were captured by 20 µl of Protein A/G agarose, followed by WB with antibodies for SFRP5 and Wnt5a, respectively.

### 2.10 Assay for luciferase activity

TOP/FOP-Flash luciferase assay was used to confirm the β-catenin-mediated transcription. TOP-Flash reporter plasmid containing multiple TCF/LEF consensus sites (Promega, Madison, WI, USA) or Renilla control plasmid (internal control) was used for transiently transfecting when cells grown to 60–70% confluence in 24-well plates, while FOP-Flash reporter plasmid containing a mutated TCF/LEF-binding site (Promega) as a negative control. After transfection for 6 h, the cells were treated with TGF-β1 (2 ng/ml) or TGF-β1 + SFRP5 (100 ng/ml), which were added to control immunoglobulin G or TGF-β1 + SFRP5 + anti-SFRP5 (1 µg/ml) medium for 72 h. A luciferase assay system (Promega) was used to determine luciferase activity. Transfection efficiency was normalized by renilla activity.

### 2.11 Statistical analyses

The results are expressed as the mean ± standard error. Differences between the control and experimental groups were examined by one-way analysis of variance and the Student–Newman–Keuls test for post hoc comparisons. SPSS software (version 20.0; IBM Corp., Armonk, NY, USA) was used for all statistical analyses. *P*-value <0.05 was considered statistically significant.


**Ethics statement:** The experimental protocol was established, according to the ethical guidelines of experimental animals and was approved by the Animal Ethics Committee of Beijing Friendship Hospital (No. YYYYDWSY220402). All methods were carried out in accordance with relevant guidelines and regulations, and the study was carried out in compliance with the ARRIVE guidelines.

## 3 Results

### 3.1 SFRP5 ameliorated RF in the UUO model

**Figure 1:**
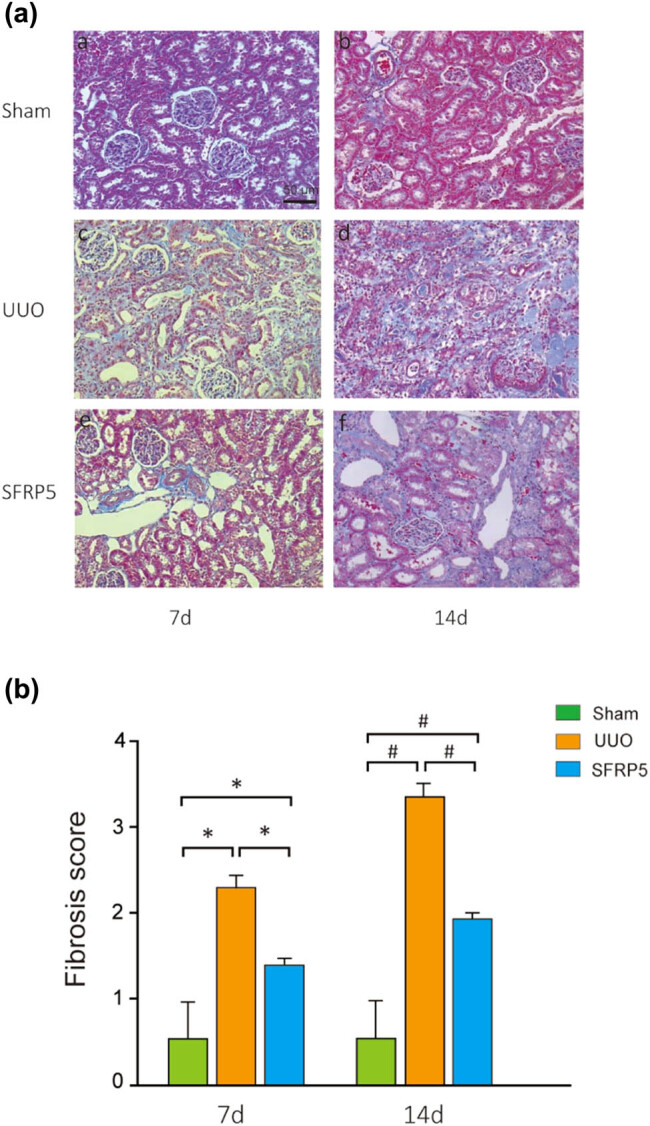
SFRP5 ameliorated RIF in a rodent model of UUO. (a) Kidney sections from various groups at 7 and 14 days after UUO were subjected to MTS. Representative micrographs (40×) showing that SFRP5 ameliorated fibrotic renal damage following obstructive injury. (b) Quantitative determination of renal fibrotic lesions (defined as percentage of the MTS-positive fibrotic area) was quantified by computer-aided morphometric analyses. Results are presented as the mean ± standard deviation per group. *P < 0.05, vs individual controls at 7 days; ^#^ *P* < 0.05, vs individual controls at 14 days.

**Figure 2:**
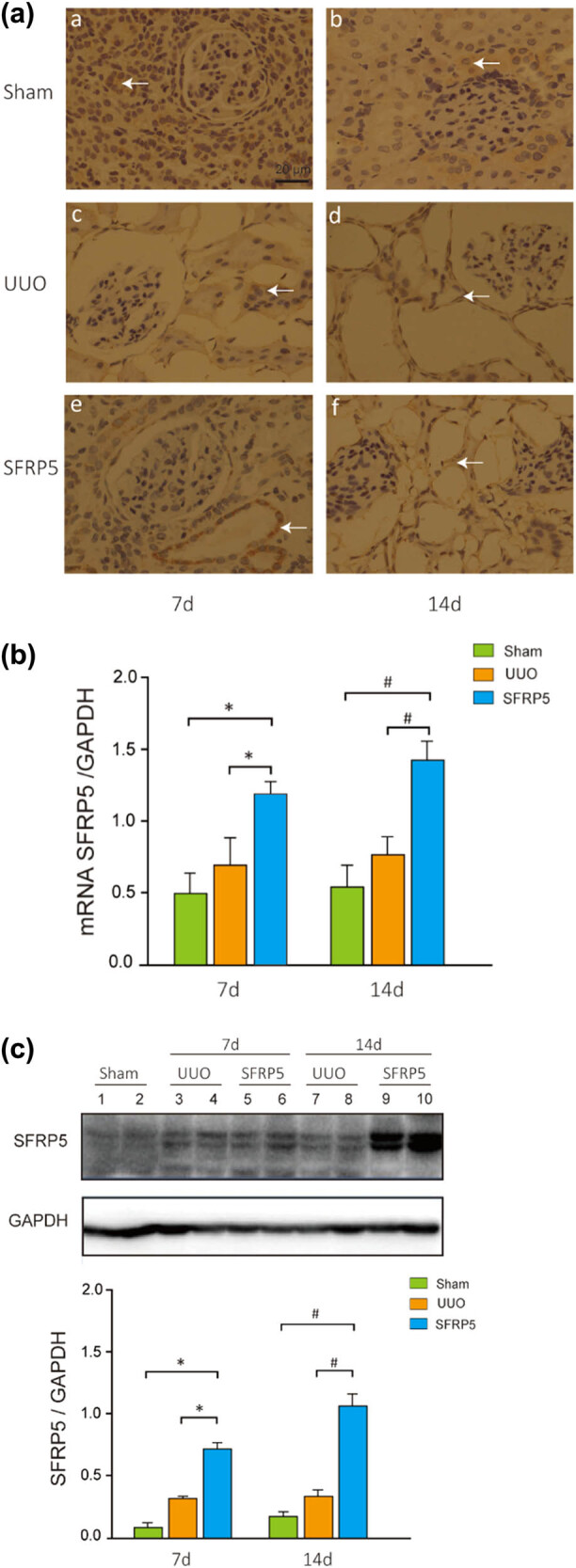
Expression of SFRP5 in a rodent model of UUO and overexpression of SFRP5 after administration of adenovirus. (a) Representative immunohistochemistry photomicrographs showing the localization and expression of SFRP5 in the ligated kidney. SFRP5 was predominantly distributed throughout the renal tubules normally. After UUO, with worsening fibrosis, SFRP5 was slightly increased. SFRP5 was significantly overexpressed in the Ad-SFRP5 group but not in the Ad-GFP (empty adenovirus) group. Arrows denote positive outcomes. Magnification: 200×. (b and c) Levels of SFRP5 mRNA, WB, and densitometric analysis of SFRP5 protein levels in each group. The results were agreement with the immunohistochemistry results. *P < 0.05, vs individual controls at 7 days; ^#^ *P* < 0.05, vs individual controls at 14 days.

After UUO, there was hypertrophy of the obstructed kidney and thinning of the renal parenchyma. At 7 days postoperatively, a large amount of patchy inflammatory cells was seen infiltrating the interstitium. There were also moderate to severe renal tubule dilatation, areas of distal tubular atrophy, swelling and degeneration of renal tubule epithelial cells, edema, and enlargement of interstitial tissue, indicating a slight proliferation of fibrosis. Moreover, prolonged obstruction caused these phenomena to worsen (Figures 1a and 2a). On MTS, there was a linear distribution of blue interstitial collagen fibers in the basement membrane of tubular in sham, which was seen to gradually increase with the passage of time for the UUO group, suggesting progressive fibrosis (Figure 1a).

To study the relation between SFRP5 and RF in UUO, we first measured the SFRP5 expression in the kidney and then used adenovirus to upregulate SFRP5 expression as described in the “Materials and Methods” section. Immunohistochemistry showed that under normal conditions, SFRP5 was predominantly distributed along renal tubules (Figure 2a). Renal expression of SFRP5 mRNA and protein was slightly increased in the UUO group and significantly overexpressed in the UUO + Ad-SFRP5 group at 7 days after UUO (*P* < 0.05) (Figure 2a–c). We reinjected adenovirus to increase the expression of SFRP5 mRNA and protein further and observe the extent of interstitial fibrosis in MTS-stained kidney tissues. RF was significantly less severe in rats with UUO treated with Ad-SFRP5 than in those treated with Ad-GFP (Figure 1a). The semi-quantitative RF score confirmed that SFRP5 protected against RF (*P* < 0.05) (Figure 1b).

### 3.2 SFRP5 inhibited EMT *in vivo*

**Figure 3:**
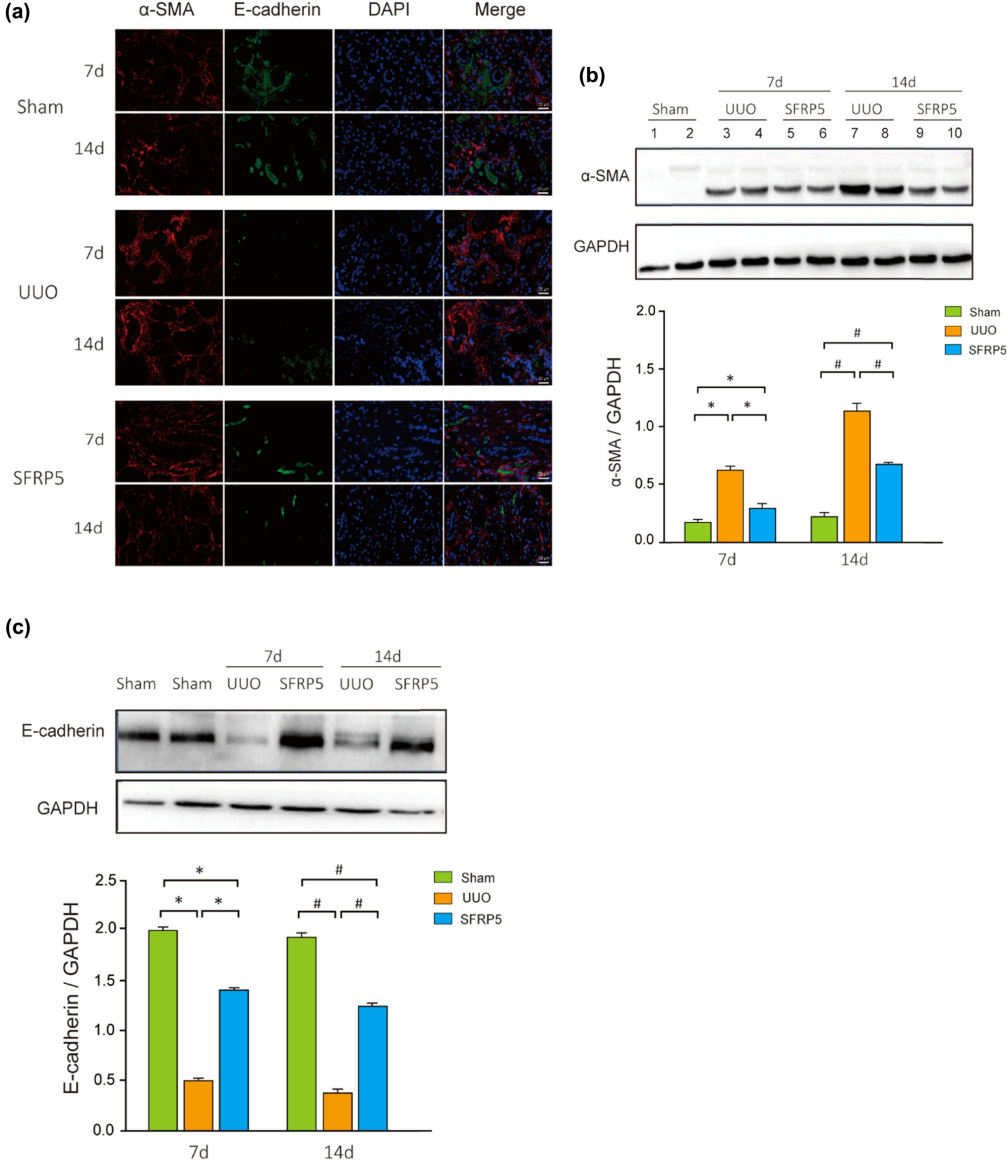
SFRP5 inhibited expression of EMT markers in a rodent model of UUO. (a) Immunofluorescence showing the changes of α-SMA and E-cadherin. Representative photomicrographs are presented. As the obstruction extended, the E-cadherin fluorescence intensity became weaker and α-SMA gradually strengthened, which was reversed by SFRP5. Magnification: 40×. (b and c) Detection by WB showed that SFRP5 inhibited α-smooth muscle actin (α-SMA) and increased E-cadherin expression at 7 and 14 days after UUO. The changes became more obvious as the fibrosis worsened. *P < 0.05, vs individual controls at 7 days; ^#^ *P* < 0.05, vs individual controls at 14 days.

In view, EMT is one of the important pathological mechanisms of RF. The EMT index was significantly greater in the surgical kidneys of the UUO group than in their counterparts treated with Ad-SFRP5 and in the sham group, indicating loss of epithelial adhesion receptor E-cadherin as well as gain of the mesenchymal marker α-SMA. The results of immunofluorescence demonstrated that E-cadherin expression decreased whereas α-SMA expression increased (Figure 3a). Specifically, the UUO group showed higher expression of α-SMA and lower in E-cadherin compared to SFRP5 and sham groups (*P* < 0.05) (Figure 3b and c). These findings were reversed by SFRP5, suggesting that EMT can be inhibited by SFRP5 *in vivo*.

### 3.3 Inhibition of the Wnt/β-catenin by SFRP5 *in vivo*

**Figure 4:**
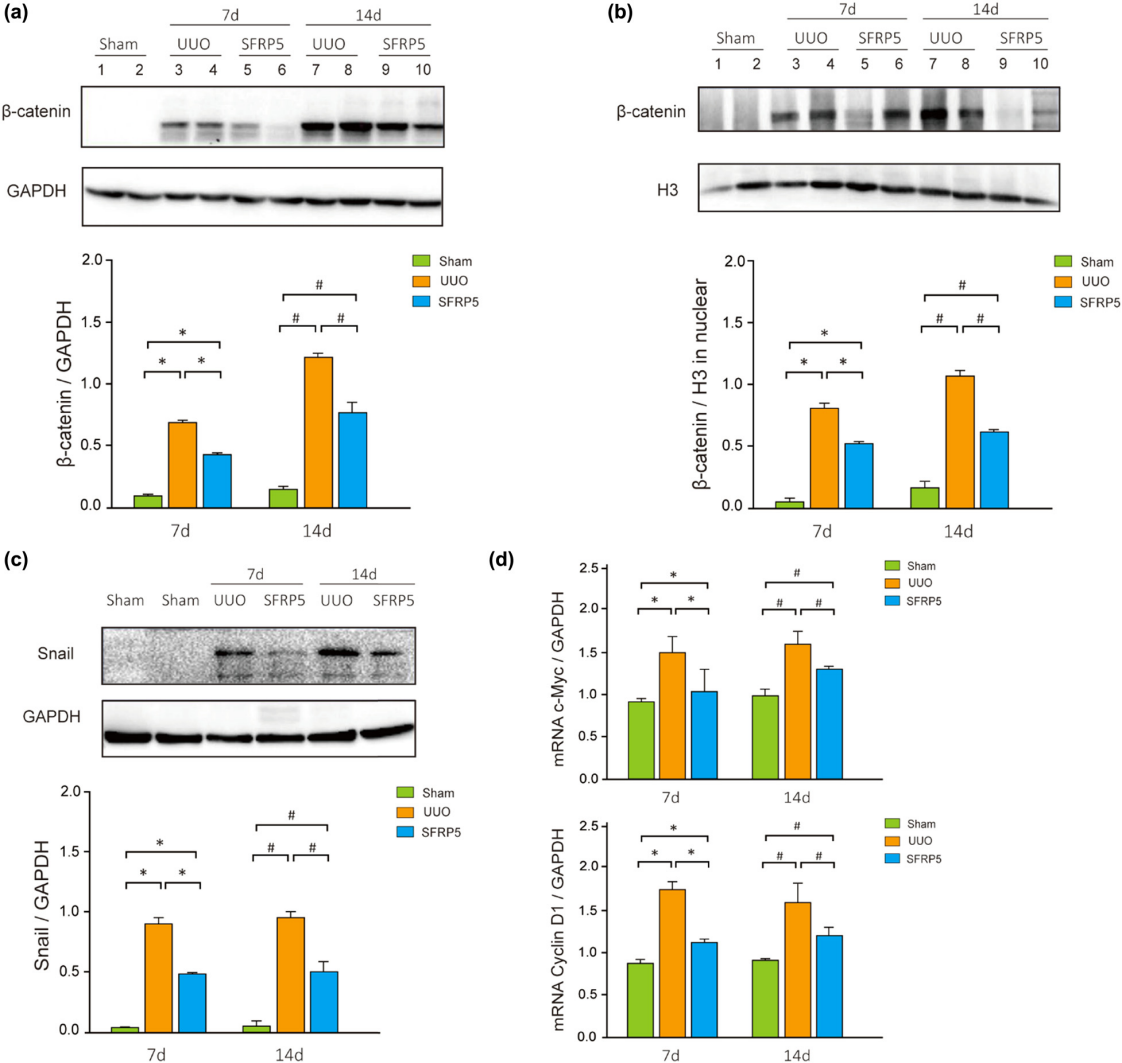
SFRP5 inhibited activation of Wnt/β-catenin in UUO-associated RIF and EMT. (a) WB revealed thatβ-catenin expression was significantly higher in UUO than in sham and could be inhibited by overexpression of SFRP5. (b) The increased β-catenin level in the nucleus of cells in the ligated kidney in the UUO groups decreased significantly after treatment with SFRP5. (c) The level of Snail protein downstream of Wnt/β-catenin increased in the UUO groups and was inhibited by SFRP5. (d) The mRNA levels of the β-catenin nuclear transcription target genes c-Myc and Cyclin D1 were consistent with the changes in β-catenin. *P < 0.05, vs individual controls at 7 days; ^#^ *P* < 0.05, vs individual controls at 14 days.

Then, the change in the Wnt/β-catenin signaling pathway in the experimental rats was explored. It had been demonstrated that activation of the Wnt/β-catenin pathway in UUO rats with RF could be inhibited by SFRP5. Compared with the sham group, the total expression of β-catenin in kidney tissue was significantly higher in the UUO (*P* < 0.05) and significantly lower in the UUO + SFRP5 group (*P* < 0.05) (Figure 4a). Next, we assessed β-catenin translocation into the nucleus via WB. As can be seen in Figure 4b, there was a significant increase in the β-catenin level in the nucleus of cells in the kidney tissue in the UUO and UUO + SFRP5 groups (*P* < 0.05). After treatment with SFRP5, the β-catenin level in the nucleus was significantly lower in the UUO + SFRP5 group (*P* < 0.05). Additionally, the expression of direct transcriptional target genes and downstream Snail protein further confirms the involvement of Wnt/β-catenin. Expression levels of the c-Myc and Cyclin D1 genes and of Snail protein were upregulated by UUO (*P* < 0.05) and suppressed by SFRP5 (*P* < 0.05) (Figure 4c and d).

### 3.4 SFRP5 inhibited EMT and fibrosis *in vitro*

**Figure 5:**
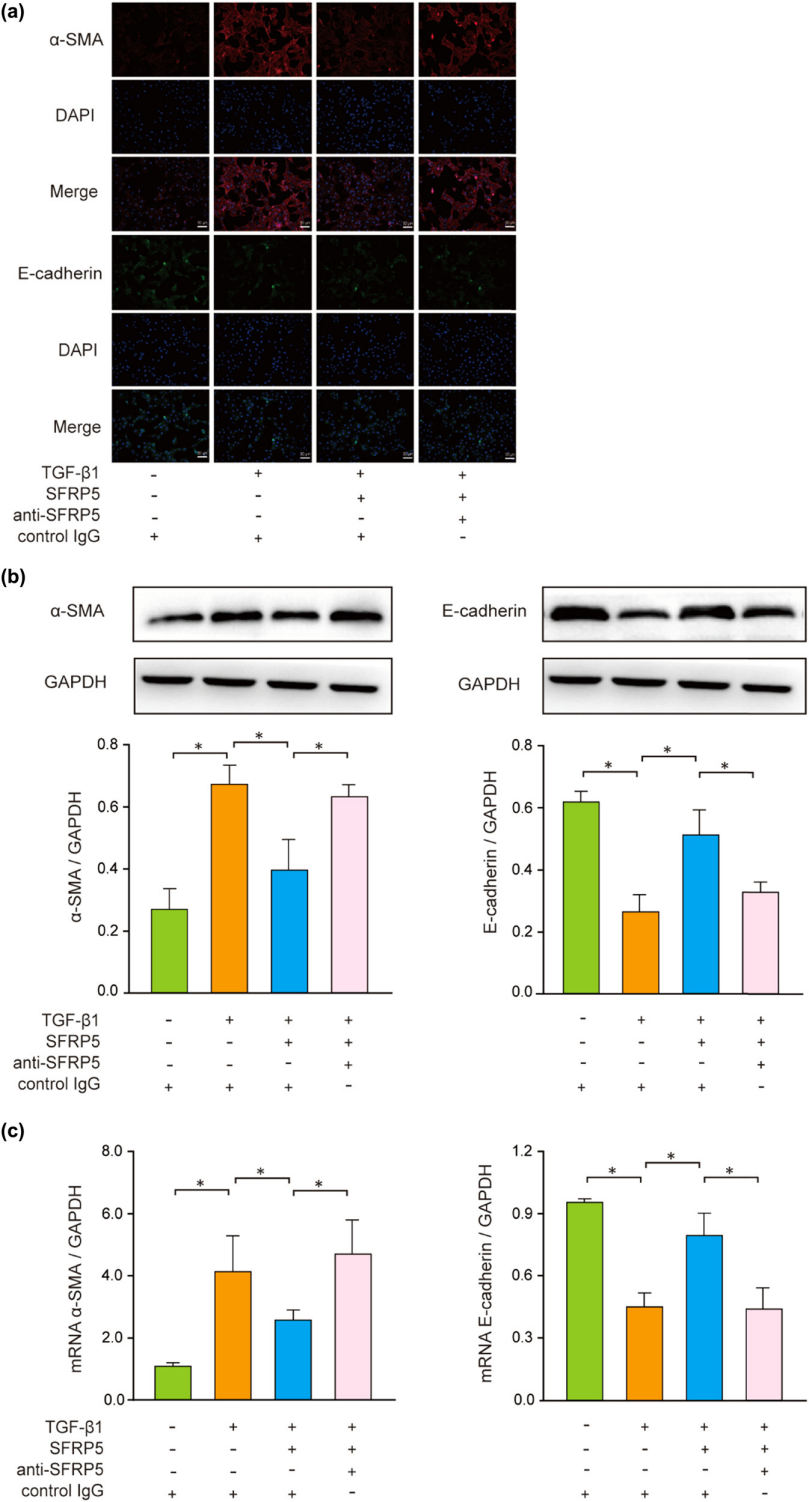

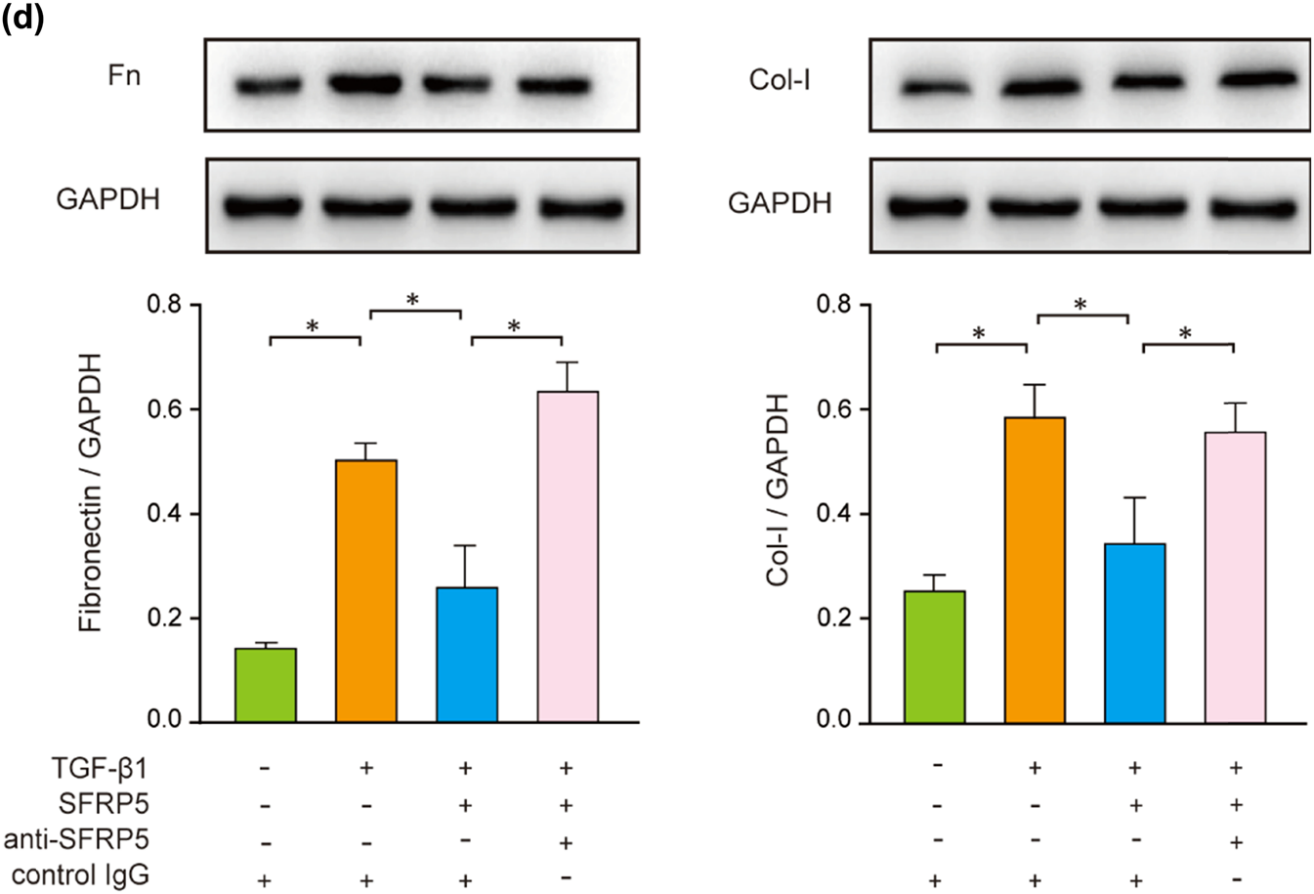
SFRP5 blocked TGF-β1-mediated EMT *in vitro*. (a) Immunofluorescence showed that SFRP5 abolished TGF-β1-induced α-SMA assembly and preserved E-cadherin integrity. Magnification: 200×. (b and d) WB demonstrated that SFRP5 reversed the increased α-SMA, collagen I, and fibronectin and decreased E-cadherin. (c) Real-time polymerase chain reaction revealed that SFRP5 reversed mRNA expression of α-SMA and E-cadherin consistent with the results of WB. Results are presented as percentages of control values after normalization to GAPDH and are the mean ± standard deviation of three independent experiments. *P < 0.05, vs individual controls.

For the proof that SFRP5 can directly act on RF, we employed the cell culture system *in vitro* where HK-2 cells were induced to undergo EMT by TGF-β1. After TGF-β1 stimulation, the immunofluorescence images directly reflected that the cells lost inherent E-cadherin instead by mesenchymal marker α-SMA. In contrast, SFRP5 abolished TGF-β1-induced α-SMA and restored E-cadherin (Figure 5a). Both WB and RT-qPCR results showed that E-cadherin expression decreased and α-SMA expression increased induced by TGF-β1, which could be obviously reversed by SFRP5 (*P* < 0.05) (Figure 5b and c). We also investigated fibronectin and collagen I to assess the fibrosis degree. In agreement with the EMT outcomes, SFRP5 alleviated TGF-β1-induced expression of fibronectin and collagen I (*P* < 0.05) (Figure 5d).

### 3.5 SFRP5 protected against EMT and RF by inhibiting the Wnt/β-catenin

**Figure 6:**
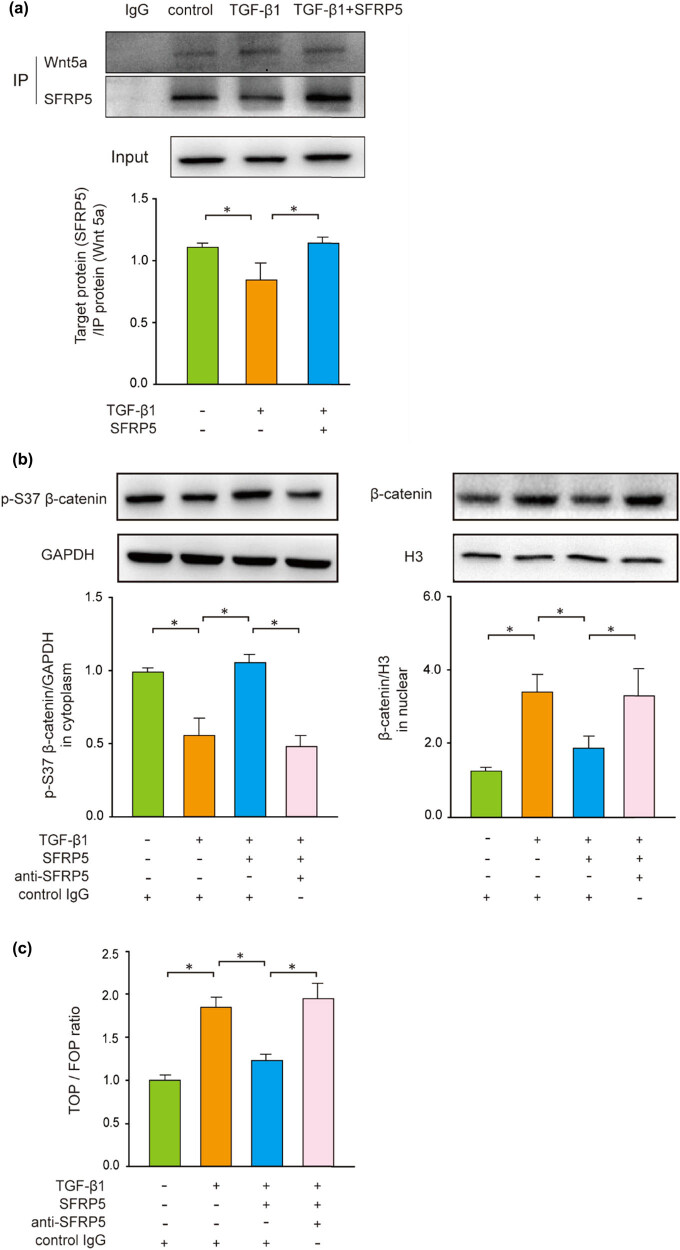

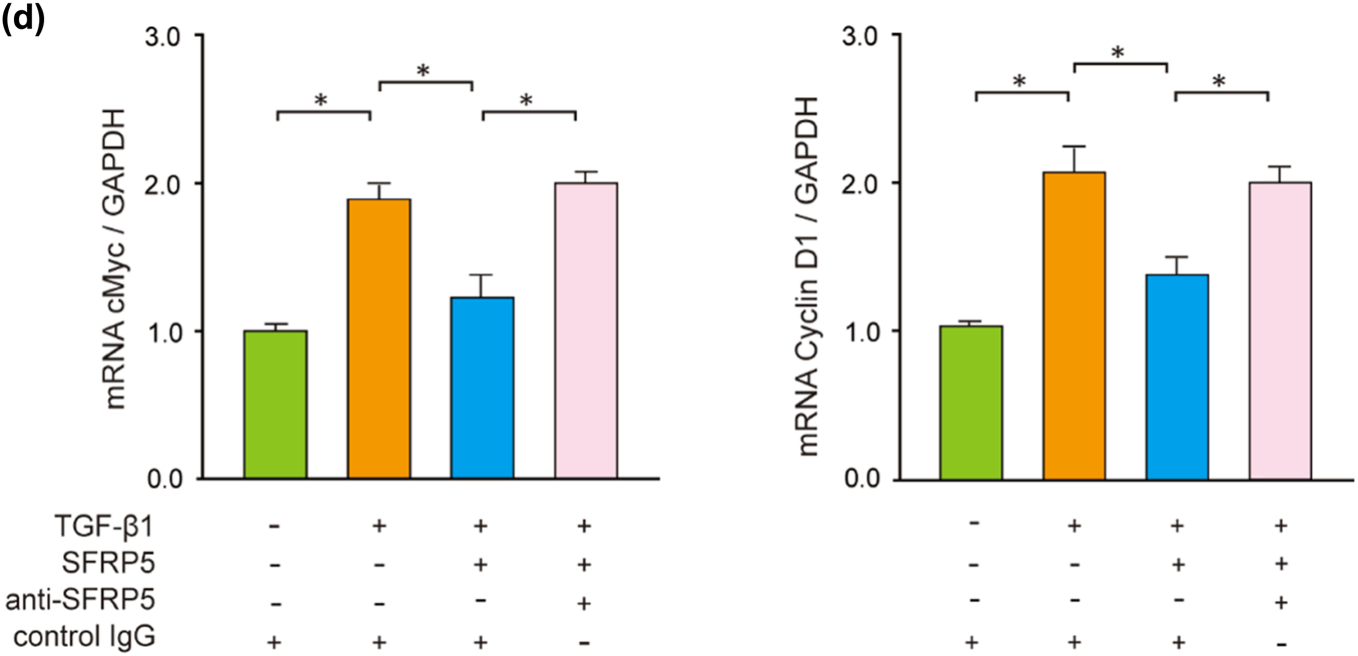
Inhibition of the canonical Wnt/β-catenin by SFRP5 during EMT and RIF *in vitro*. (a) Immunoprecipitation analysis revealed that SFRP5 inhibited the TGF-β1-induced decreased ability of SFRP5 to bind to Wnt5a. (b) The level of cytoplasmic phospho-β-catenin (S37) and nuclear localization of β-catenin was assessed with WB normalized by GAPDH or H3. SFRP5 reversed the decreased level of p-S37 β-catenin in the cytoplasmic lysate and the increased nuclear β-catenin induced by TGF-β1. Quantification was carried out by measuring the integrated optical density. Representative images from three separate experiments are shown. (c) TOP/FOP-Flash luciferase assay showed upregulating β-catenin-dependent gene transcription, which could be inhibited by SFRP5 during EMT in TGF-β1-treated cells. (d) SFRP5 attenuated TGF-β1-induced activation of the β-catenin nuclear-transcriptional target genes c-Myc and Cyclin D1. Data are shown as the mean ± standard deviation of the mean of three independent experiments. *P < 0.05, vs individual controls.

First, the incubation with TGF-β1 reduced the binding of SFRP5 to Wnt5a via immunoprecipitation assay, and the inhibition of TGF-β1 could be reversed by the supplement of SFRP5 protein (Figure 6a). In addition, WB was used to evaluate the phosphorylation and nuclear translocation of β-catenin. When cells were incubated in a TGF-β1 medium, the β-catenin phosphorylation site at the residue S37(p-S37 β-catenin) was shown to be restricted in the cytoplasmic lysates (*P* < 0.05); thus, there was an increase in nuclear β-catenin (*P* < 0.05) (Figure 6b). The addition of SFRP5 resulted in a marked decrease in nuclear β-catenin (*P* < 0.05) and a restorative increase in p-S37 β-catenin (*P* < 0.05), both were reversed by anti-SFRP5 (*P* < 0.05).

We then evaluated the transcriptional activation of β-catenin-dependent directly by nucleofection in TCF/LEF. As can be seen in Figure 6c, after treatment with TGF-β1, TCF/LEF luciferase activity, which represents activation of Wnt/β-catenin, showed an increase of approximately two-fold compared with the control (*P <* 0.05). But this increase could be attenuated by the addition of SFRP5 (*P* < 0.05). The Wnt/β-catenin direct transcriptional target genes were also assessed to further determine the specific participation of Wnt/β-catenin signaling. The genes of c-Myc and Cyclin D1 were upregulated by the addition of TGF-β1 (*P* < 0.05), which was repressed by SFRP5 and counteracted by anti-SFRP5 (*P* < 0.05) (Figure 6d).

## 4 Discussion

Progressive RF is the major pathological hallmark of most forms of CKD [17,18]. Current therapies have limited efficacy and new agents are needed to slow the progression of CKD by preventing RF. In this study, a rodent model of RF was established by UUO. After 7 days, the EMT index increased for α-SMA and decreased for E-cadherin in the ligated kidney tissue, suggesting that EMT had occurred. There was a marked increase in the volume of the lateral kidney tissue and the cortex became thinner. Pathological examination showed that a large amount of collagen fibers had been deposited in the interstitium. As in a previous study [19], these changes progressively worsened over time. In other studies, pathological examination of related signaling pathways found that abnormal activation of the Wnt/β-catenin is involved in the RF induced by UUO [20,21], and obstruction could lead to the aggregation of β-catenin in both the cytoplasm and nucleus of renal tubular epithelial cells and activation of the canonical Wnt pathway. *In vivo*, we found a significant increase in the expression of β-catenin both in the tissues and nuclei, suggesting that the Wnt/β-catenin signaling pathway was abnormally activated during the EMT and RF induced by UUO.

SFRPs have homology with specific frizzled protein receptors in the Wnt signaling pathway and can bind with Wnt protein ligands or frizzled receptors, thereby inhibiting activation of Wnt signaling pathway [22]. The preliminary research found that intraperitoneal injection of recombinant SFRP4 in UUO mice inhibited the Wnt/β-catenin pathway and lessened the severity of RF [23]. The authors of that report concluded that β-catenin signaling is activated in tubular epithelial and interstitial cells following renal injury and that recombinant SFRP4 interfered with de-differentiation of epithelial cells and with differentiation and function of fibroblasts during progression of RF. Other researchers identified that SFRP1 regulated the progression of RF in the UUO mouse model. The degree of RF was found to be significantly worse in SFRP1 gene knockout (SFRP1−/−) mice after UUO than in wild-type mice [24]. Moreover, the results of mechanistic research indicate that SFRP1 was required for inhibition of renal damage through the non-canonical Wnt/PCP pathway but not the canonical Wnt/β-catenin [24].

Expression of SFRP5 in the kidney is second only to that in white adipose tissue in mice [9]. Recently, Yu et al. found that indoxyl sulfate promotes RF by inducing DNA hypermethylation of SFRP5 followed by activated Wnt/β-catenin [12]. Therefore, we speculated that SFRP5 may alleviate RF by directly inhibiting Wnt/β-catenin. In a rodent model of UUO, there was a slight compensatory increase in renal expression of SFRP5. To examine if this change in SFRP5 was related to the occurrence of RF, the adenovirus amplification was used to increase expression of SFRP5 in kidney tissues. We found that SFRP5 prevented RF and EMT. Compared with the UUO group, there was a decrease in the expression of EMT-related indices as well as the degree of RF, suggesting that overexpression of SFRP5 can slow the course of RF. After SFRP5 became overexpressed, there were marked decreases in total β-catenin levels in kidney tissue, the amount of activated β-catenin in the nucleus, and the level of Snail protein downstream. These findings are consistent with those of a previous study, which found that a β-catenin transcription inhibitor reduced expression of downstream target genes, such as those for Snail, collagen I, and α-SMA, and decreased RF in UUO model [25]. Snail downstream of the β-catenin pathway can inhibit transcription and expression of E-cadherin, resulting in the loss of tight junctions between epithelial cells, mediating the transformation of epithelial cells into mesenchymal cells, and promoting RF [26].

To further explore putative cellular mechanisms underlying RF and EMT regulation by SFRP5, we assessed the signaling pathways involved. The canonical Wnt/β-catenin pathway has already been shown to be activated in the development of RF [27,28,29]. The aim of the current study was to investigate whether Wnt/β-catenin is regulated by SFRP5 *in vitro* and *in vivo*. We had previously shown that TGF-β1 induces nuclear translocation of β-catenin *in vitro*, with subsequent activation of an β-catenin-dependent TCF/LEF promoter. Furthermore, we found that TGF-β1 caused increased cytosolic β-catenin accumulation by negatively regulating β-catenin phosphorylation, which was first shown to occur at S37. The enhanced expression of downstream target genes also confirmed the activation of Wnt/β-catenin. These findings suggest that SFRP5 can reduce EMT and slow the course of fibrosis in the renal interstitium by inhibiting abnormal activation of the Wnt/β-catenin. Though the influence of the non-canonical Wnt pathway was not explored, we did use neutralizing antibody to verify the specificity of SFRP5 for Wnt/β-catenin inhibition. Therefore, we believed that downregulation of Wnt/β-catenin elicited by SFRP5 can attenuate the RF.

There were still some limitations here, which did not check the effect of SFRP5 on the other Wnt signaling. Therefore, further studies are required to focus on the non-canonical Wnt pathway meanwhile the crosstalk between them. More studies are needed to determine the effect of SFRP5 on ligand proteins in the Wnt signaling pathway during RF and whether a non-canonical Wnt pathway is involved.

## 5 Conclusion

Our findings suggest that the antifibrotic effects of SFRP5 are mediated by antagonism of the Wnt/β-catenin signaling pathway, resulting in decreased production of ECM and improvement in tubulointerstitial lesions. SFRP5 may be able to suppress RF and is a potential therapeutic target for the prevention or treatment of CKD.
